# Targeting of VPS18 by the lysosomotropic agent RDN reverses TFE3-mediated drug resistance

**DOI:** 10.1038/s41392-021-00547-x

**Published:** 2021-06-07

**Authors:** Huanmin Niu, Lilin Qian, Yanhai Luo, Fang Wang, Hongbo Zheng, Yanhui Gao, Hanbo Wang, Xuelei Hu, Huiqing Yuan, Hongxiang Lou

**Affiliations:** 1 Key Laboratory of Natural Products & Chemical Biology of Ministry of Education, Institute of Medical Sciences, The Second Hospital, Cheeloo College of Medicine, Shandong University, Jinan, China; 2 Department of Natural Product Chemistry, School of Pharmaceutical Sciences, Cheeloo College of Medicine, Shandong University, Jinan, China; 3 Minimally Invasive Urology Center, Shandong Provincial Hospital, Jinan, China; 4 Department of Thoracic Surgery, Qilu Hospital, Cheeloo College of Medicine, Shandong University, Qingdao, China

**Keywords:** Cancer therapy, Target identification

**Dear Editor,**

Multidrug resistance (MDR) is still a major challenge for successful cancer treatments. Numerous mechanisms that confer therapy-induced drug resistance have been extensively investigated to explore how to combat the MDR. In this regard, lysosomal sequestration has demonstrated to be a mechanism contributing to drug resistance via an “off-target” effect in which hydrophobic and weakly basic chemotherapeutic agents are trapped in lysosomes, sequestering them from their targets.^1^ Approaches that disrupt lysosomal acidification, modulate acid sphingomyelinase (ASM), and increase lysosomal membrane permeabilization were developed to overcome drug resistance.^2^ Recently transcription factor E3 (TFE3) and TFEB are emerged as master regulators of lysosomal biogenesis and autophagic process in response to cellular stresses, including therapeutic treatments, interruption of TFE3/TFEB-mediated effects therefore has great therapeutic potential in cancer.^3^

In the present study, we reveal a novel mechanism by which TFE3/lysosome activation in response to therapeutics, such as docetaxel (Doc) contributes to MDR via promoting lysosomal localization of multidrug resistance-associated protein 2 (MRP2) and enhancing drug sequestration in lysosomes. We also identified the lysosomal vacuole protein sorting 18 (VPS18) as a potential target for the reversal of chemoresistance by a novel lysosomotropic agent RDN, an aminomethylated derivative of naturally occurring bisbibenzyl riccardin D.

We initially assess the changes in gene expression in Doc-induced multidrug-resistant PC3/Doc cancer cells. The expression profiles of genes related to metabolism in lysosomal and mitochondrial pathways were particularly altered in resistant cells (Fig. 1a and Supplementary Fig. 1a). Examination of lysosome-associated parameters demonstrated that the numbers of lysosomes and autophagosomes were noticeably increased in different resistant cell lines (Supplementary Fig. 1b–h). Consistent with the observations in cultured cells, an increase in the expression of lysosome genes was clearly shown in tumors treated with Doc, vinblastine (VCR), doxorubicin (Dox), and etoposide (VP16) (Supplementary Fig. 1i–l). Thus, lysosome activation is an outcome of treatments with different antitumor agents.

**Fig. 1:**
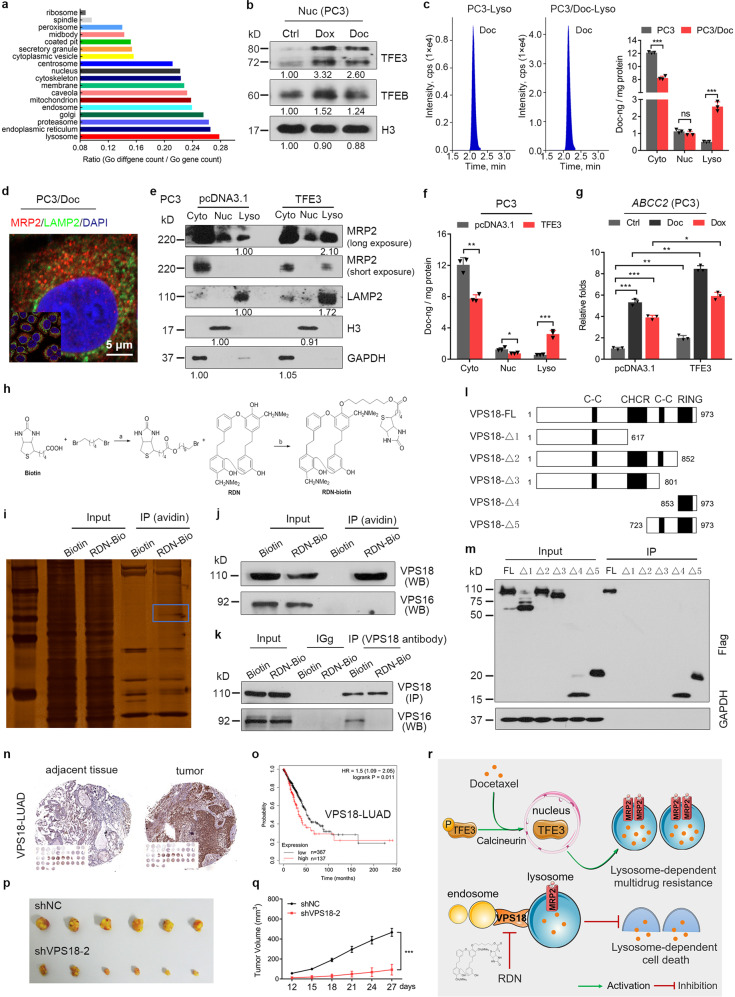
**a** GO analysis of organelle-related genes according to cellular component. **b** The nuclear (Nuc) fractions of TFEB-expressing and TFE3-expressing PC3 cells treated with Doc (12 nM). Doxorubicin (Dox, 0.8 μM) was used as a positive control. **c** The Doc concentration in cytoplasm (Cyto), nuclear (Nuc), and lysosome (Lyso) was measured by high-performance liquid chromatography (HPLC). **d** Distribution of MRP2 and its colocalization with LAMP2 in PC3/Doc cells. **e** The expression of MRP2 and typical markers were detected by western blotting in TFE3-overexpressing cells. **f** Changes in the distribution of Doc in different organelles of TFE3-overexpressing cells. **g** Cells overexpressing TFE3 were treated with a combination of Doc (12 nM) and Dox (0.8 μM), and the expression of *ABCC2* was analyzed by qPCR. **h** The synthesis of RDN-biotin, a chemical probe of RDN. **i** RDN-Bio (1 μM) interactors purified by streptavidin affinity purification were separted by SDS-PAGE. **j** Western blotting showing the coimmunoprecipitation of RDN-Bio (1 μM) with VPS18 but not with VPS16 and Biotin. **k** The interaction between VPS18 and VPS16 was disrupted by RDN-Bio (1 μM). **l** Model of human VPS18 mutants lacking three domains in the C-terminus (VPSΔ1), one domain in the C-terminus (VPSΔ2), two domains in the C-terminus (VPSΔ3), the RING domain (VPSΔ4), or the RING domain and the CC domain (VPSΔ5). **m** Western blotting showing the coimmunoprecipitation of RDN-Bio with overexpressed Flag-tagged full-length RDN, or the RING domain (VPSΔ4) or the RING with the CC domain (VPSΔ5) in 293T cells. **n** The expression of VPS18 in lung cancer (LUAD) and adjacent noncancerous tissues was detected by immunohistochemistry. **o** VPS18 is correlated with poor prognosis in human lung cancer. **p** Photographs of excised tumors from two groups (PC3/Doc-shNC and PC3/Doc-shVPS18-2) are shown. **q** Tumor volumes in different groups were recorded every 3 days. **r** The working model. TFE3/lysosome activation induced by therapeutics such as docetaxel (Doc) contributes to MDR by promoting lysosomal localization of MRP2 and enhancing drug sequestration in lysosomes. Lysosomal VPS18 as a potential target for the reversal of chemoresistance by a novel lysosomotropic agent RDN. Data are the mean ± SD; *p < 0.05, **p < 0.01, and ***p < 0.001

Given the importance of TFEB and TFE3 in the regulation of lysosomal biogenesis,^3^ we therefore determined the involvement of these two factors in the therapy-induced metabolic shift to lysosomes. The results clearly showed that clinical drugs significantly activated TFEB and TFE3, particularly TFE3, promoting TFE3 nuclear translocation (active form with hypro-phosphorylation) in vitro and in vivo (Fig. 1b and Supplementary Fig. 2a–e). We also found that TFE3 activation, at least partially, was induced by calcineurin in response to chemotherapeutics, because specific inhibition of calcineurin by FK520 could reduce the drug-driven nucleation of TFE3 (Supplementary Fig. 2f–j).

We next determined whether therapy-activated TFE3/lysosome contributes to drug resistance. TFE3 downregulation remarkably restored the sensitivity of PC3/Doc cells to either Doc or Dox, while the overexpression of TFE3 significantly increased the tolerance of PC3 cells to the drugs (Supplementary Fig. 3a–h), indicating a role of TFE3 in conferring MDR. Moreover, as measured by high-performance liquid chromatography (HPLC), there was significantly less Doc and Dox in the cytoplasm, whereas high levels of trapped Doc and Dox were detected in the lysosomes of resistant cells (Fig. 1c and Supplementary Fig. 3i–k). Thus, lysosome-mediated Doc or Dox trapping causes off-target effects, thereby reducing their antitumor efficacy.

To explore the mechanisms underlying lysosomal drug sequestration, we tested whether ABC family transporters, which act as a cellular membrane pumps and are critical in mediating MDR. Screening assays led us to identify MRP2 (*ABCC2*) as an important mediator in lysosomal drug resistance (Supplementary Fig. 4a–d). Although MRP2 mainly localizes on the cellular membrane, a significant amount of MRP2 was present in the cytoplasm and shown to co-localize with lysosomal-associated membrane protein-2 (LAMP2) in resistant cells (Fig. 1d). Moreover, TFE3 stimulated MRP2 migration to lysosomes and enhanced lysosomal drug trapping (Fig. 1e, f and Supplementary Fig. 4e), whereas, genetic inhibition of MRP2 significantly suppressed TFE3-mediated drug resistance (Supplementary Fig. 4f). Further investigations identified MRP2 as a target of the TFE3, which could transcriptionally augment MRP2 expression via an E-box motif in MRP2 promoter upon chemical treatments (Fig. 1g and Supplementary Fig. 4g–i). Thus, therapy-driven TFE3 activation stimulated MRP2 expression and localization in lysosomes, boosting lysosome-mediated drug trapping and resistance.

We next determine whether target the TFE3/lysosome axis is effective in reversal of MDR. As TFE3 is a critical factor in controlling numerous gene expressions, we sought to use a small molecule RDN that acted selectively towards lysosomes,^4^ rather than TFE3, and exerted more potential inhibition on resistant cells than that of hydroxychloroquine (HCQ) and salinomycin (SAL), two well-documented lysosome target agents (Supplementary Fig. 5a–e). Notably, RDN had no obvious effect on MRP2 expression but disrupted MRP2 lysosomal localization (Supplementary Fig. 5f, g), and exerted the potent antitumor efficacy against resistance with no detectable toxicity in vitro and in vivo assays, while Doc was less effective (Supplementary Fig. 5h–k). To define the molecular target of RDN in the lysosome, a chemical probe, RDN-biotin, was designed to allow purification of RDN partners (Fig. 1h and Supplementary Fig. 5l, m). After the incubation of cell lysates with RDN-Bio or biotin alone, a specific band in precipitated RDN-Bio-streptavidin complexes was subjected to proteomic analysis (Fig. 1i). Putative RDN-biotin-interacting partners identified by mass spectrometry were vacuole protein sorting 18 (VPS18), ATP binding cassette sub-family F member 1, heat shock protein 105 kDa, and heat shock 70 kDa protein 4. Considering the observed dependence of resistance on the lysosome, we focused our attention on VPS18, a central member of the VPS-C core complex that plays a pivotal role in lysosomal maturation.^5^ As shown in Fig. 1j, VPS18, but not VPS16 (one VPS-C partner of VPS18) and control biotin, was shown to co-precipitate with RDN-Bio. RDN disrupted VPS18 function, as evidenced by the loss of the association between VPS18 and VPS16 (Fig. 1k). Furthermore, the RING domain in VPS18 is essential for RDN targeting after analysis of interactions between VPS18 deletion mutants and RDN (Fig. 1l, m). These results demonstrate that RDN binds to the RING domain of VPS18, leading to disruption of the VPS-C core complex and lysosomal function.

To explore whether VPS18 is a significant pathological effector, we firstly detect the level of VPS18 in cancers. The results indicated that VPS18 expression in several cancers was higher than that in adjacent noncancerous tissues, including prostate cancer (PRAD), bladder cancer (BLCA), liver cancer (LIHC), and lung cancer (LUAD) (Fig. 1n and Supplementary Fig. 6a). A high level of VPS18 is correlated with poor prognosis in malignant cancers (Fig. 1o and Supplementary Fig. 6b). VPS18 knockdown significantly inhibited cancer cell proliferation and tumor growth in mice (Fig. 1p, q and Supplementary Fig. 6c–g). Importantly, we observed that VPS18 expression was inducible by different chemotherapeutic agents and dramatically increased in drug resistant cells (Supplementary Fig. 6h–j). VPS18 silencing significantly increased the sensitivity of resistant cells to Doc and Dox, while the overexpression of VPS18 inhibited the efficacy of chemotherapeutics on cell proliferation (Supplementary Fig. 6k, l). Therefore, VPS18 emerges as a novel molecular indicator of tumor prognosis and a target in reversing lysosome-mediated resistance.

In summary, we provided evidence demonstrating a novel function of TFE3/lysosome/MRP2 in the development of MDR via the lysosome sequestration mechanism. The contribution of lysosomal VPS18 to the development of cancer and drug resistance could be explored as a potential therapeutic target, which is specifically inhibited by a novel compound RDN to treat acquired resistant cancers (Fig. 1r).

## Supplementary information

Supplementary-R2,Clean version
