# Supplementary material for: Targeting of VPS18 by the lysosomotropic agent RDN reverses TFE3-mediated drug resistance
Source: Signal Transduct Target Ther. 2021 Jun 7;6:224. doi: 10.1038/s41392-021-00547-x (PMC8184988; doi:10.1038/s41392-021-00547-x)
Supplement: Supplementary file 1 — Supplementary-R2,Clean version [file 41392_2021_547_MOESM1_ESM.docx]

**Supplementary Materials**

**Targeting of VPS18 by the lysosomotropic agent RDN reverses TFE3-mediated drug resistance**

Huanmin Niu^a^, Lilin Qian^b^, Yanhai Luo^a^, Fang Wang^a^, Hongbo Zheng^b^, Yanhui Gao^b^, Hanbo Wang^c^, Xuelei Hu^d^, Huiqing Yuan^a^*, Hongxiang Lou^b^*

Correspondence to:

*Huiqing Yuan (Email: lyuanhq@sdu.edu.cn)

*Hongxiang Lou (Email: louhongxiang@sdu.edu.cn.)

**This file includes:**

Author Contributions

Acknowledgements

Availability of data

Materials and Methods

References

Figures S1 to S6

Tables S1 and S2

**Author Contributions**

Huanmin Niu carried out experiments, made figures and wrote the manuscript; Lilin Qian, Yanhai Luo, Hongbo Zheng, Fang Wang, Yanhui Gao performed some of experiments including cell culture, animal trials, and HPLC analysis; Hanbo Wang, Xuelei Hu analyzed clinical data; Huiqing Yuan, Hongxiang Lou supervised and designed the research, analyzed and interpreted the data, revised and polished the manuscript.

**Acknowledgements**

We thank Dr. Yoshihiro Kawaoka (University of Tokyo) for kindly providing us the full-length VPS18 and truncated VPS18 plasmids. This work was supported by the National Natural Science Foundation of China (81872896, 81473238, 81874293), the Shandong Key Innovative Research Program (2018CXGC1216), and the PCSIRT (IRT-17R68).

**Availability of data**

The datasets used and/or analyzed during the present study are available from the corresponding author on reasonable request.

**Materials and Methods**

**Cell culture and treatments**

Prostate cancer (PCa) PC3 cells (the Cell Bank of the Chinese Academy of Sciences, Shanghai, China) and docetaxel-resistant PC3/Doc cells, were cultured in F12K medium supplemented with 10% fetal bovine serum (HyClone) and 100 U/ml penicillin and 100 g/ml streptomycin. H460 and taxol-resistant H460/Tax lung adenocarcinoma cells, murine RM1 and docetaxel-resistant RM1/Doc PCa cells were cultured in RPMI-1640 medium supplemented with 10% fetal bovine serum (HyClone) and antibiotics (penicillin and streptomycin). KB, vincristine-resistant KB/VCR cells and 293T cells were cultured in DMEM (HyClone). The drug resistant cell lines were routinely developed.^1^ Briefly, drugs were added to the medium of parental cells at gradually increasing concentrations from IC10 to IC50. During the continuous exposure, the drugs medium was replaced with fresh medium containing the desired concentration of drugs every 3 days and maintained at this concentration for at least 4 generations. Cells survived at concentrations up to IC50 were named resistant cells. All these cells were routinely cultured in 5% CO_2_ at 37 °C. After chemical treatments, cells were collected for western blotting or other assays. DMSO was used as the vehicle control. The experiments were performed in duplicate and repeated three times.

**Reagents**

The structure of RD-N, an aminomethylated derivative of riccardin D, was identified as reported previously.^2^ Docetaxel (Doc), doxorubicin (Dox), vincristine (VCR), and etoposide (VP16) were purchased from the Second Hospital of Shandong University. Hydroxychloroquine sulfate (#S4430) and FK520 (#S7411) were purchased from Selleck. Salinomycin (#HY-17439) was purchased from MedChem Express. Rapamycin (#553210) was purchased from Sigma-Aldrich. Lyso-Tracker (#C1046), DAPI (#C1005), Hoechst33342 (#C1026), [Rhodamine](javascript:;) (Rho) (#C2007), and a Reactive Oxygen Species Assay Kit (#S0033) were purchased from Beyotime. D-Luciferin potassium salt was purchased from Invitrogen (#L2916). A Calcineurin Cellular Activity Assay Kit (#KL-CaN-Hu) was purchased from KALANG. AN Amplite™ Fluorimetric Acidic Sphingomyelinase Assay Kit (#13622) was purchased from AAT Bioquest.

**Gene expression profiling by Affymetrix microarray**

To compare the gene expression in PC3 and PC3/Doc cells, the cells were harvested and used for transcriptome profiling using an Affymetrix microarray. Gene expression data were analyzed using an Affymetrix Expression Console™ software.

**Transmission electron microscopy (TEM)**

To visualize cellular ultrastructure by electron microscopy, cells were harvested by centrifugation. The cell pellet was resuspended in 10 ml of ice-cold 3 % glutaraldehyde fixative in 0.1 M sodium cacodylate buffer for 35 min at 4 °C. The cells were then collected by centrifugation and resuspended in 1 ml of ice-cold 0.2 M sodium cacodylate buffer. The samples were then sent for visualization by electron microscopy.

**Western blot analysis and antibody**

After treatment with RDN and other compounds, cells were washed with ice-cold PBS and lysed in RIPA lysis buffer containing a fresh protease and phosphate inhibitor mixture (50 mg/ml aprotinin, 0.5 mM phenylmethanesulfonyl fluoride, 1 mM sodium orthovanadate, 10 mM sodium fluoride and 10 mM β-glycerolphosphate). Cell lysates were then prepared for Western blotting. Protein concentrations were quantified by BCA protein assay. Blots were incubated with primary antibodies against P62 (Proteintech, [18420-1-AP](http://www.ptgcn.com/products/SQSTM1-Antibody-18420-1-AP.htm)), LC3 (abcam, ab51520), CTSB (abcam, ab58802), LAMP2 (Santa Cruz, Sc-71492), RAB5 (Cell Signaling Tech, 2143), β-Actin (Santa Cruz, Sc-47778), VPS18 (abcam, ab178416; Proteintech, 10901-1-AP), TFEB (Cell Signaling Tech, 4240), TFE3 (Cell Signaling Tech, 14779), Histone H3 (Cell Signaling Tech, 4499), Flag (Sigma-Aldrich, F1804), p-mTORC1 (Cell Signaling Tech, 2971), p-P70S6K (Cell Signaling Tech, 9205), p-GSK3β (Cell Signaling Tech, 9315), p-AKT (Cell Signaling Tech., 4058), CaN A (Cell Signaling Tech, 2614), RAB7 (Cell Signaling Tech, 9367), MRP2 (Sigma-Aldrich, M8316), Biotin (abcam, ab1227), VPS16 (Proteintech, 17776-1-AP), NBR1 (Proteintech, 160041-1-AP), CALCOCO2 (Proteintech, 12229-1-AP), ATG5 (Cell Signaling Tech, 9980), LAMP1 (Cell Signaling Tech, 9091) and GAPDH (Santa Cruz, Sc-32233) overnight at 4°C prior to being probed with the appropriate peroxide-conjugated secondary antibodies.

**Real-time quantitative PCR**

Total RNA was extracted using a TRIzol Kit. cDNA was prepared using a Prime Script RT [Reagent](https://www.sciencedirect.com/topics/medicine-and-dentistry/reagent) Kit with gDNA Eraser. Real-time quantitative PCR (qPCR) was performed with SYBR Green. The primers used to amplify target genes are listed in Table S1.

**Analysis of lysosome-related genes and the antitumor efficiency of RDN and VPS18 in drug resistant animal models**

C57BL/6 mice (6 weeks old) were obtained from the Animal Center of the China Academy of Medical Sciences (Beijing, China). Murine RM1 PCa cells were injected into the right flanks of the mice and allowed to establish tumors. When the tumors reached 50~100 mm^3^, the mice were given the first-line clinical chemotherapeutics docetaxel (5 mg/kg), doxorubicin (2 mg/kg), vincristine (0.5 mg/kg), etoposide (10 mg/kg),^3, 4, 5, 6^ by intraperitoneal injection every 2 days 7 times. Tumor samples in medium were disrupted with sterile blades to produce a cell suspension. The cells were cultivated, and their resistance was confirmed by MTT assay. The resistant cells were then used to infect mice, which were treated with chemotherapeutic drugs for the next course of chemotherapy.

Male BALB/c nude mice (6 weeks old) were obtained from the Animal Center of the China Academy of Medical Sciences (Beijing, China). Human PCa resistant cells were then infected with luciferase virus to obtain PC3/Doc-Luc cells. PC3/Doc-Luc cells were routinely injected into the left flanks of the mice and allowed to develop tumors. After the tumors reached 50~100 mm^3^, the animals were randomly assigned to different groups (n=5) and treated with 20 mg/kg RDN, 5 mg/kg Doc. The drugs were injected every 2 days for 2 weeks. The tumor volumes and animal weights were recorded. Tumor sizes were visualized on an IVIS imaging system (Caliper Life Sciences USA). Living Image 3.1.0 software (Caliper Life Science, USA) was used to assess the tumors by photometry. Tumor volumes (mm^3^) were calculated from the formula 0.5×L×W^2^ (L=length, W=width).

Male BALB/c nude mice (6 weeks old) were obtained from the Animal Center of the China Academy of Medical Sciences (Beijing, China). Human PCa PC3/Doc-shNC or PC3/Doc -shVPS18 cells were injected into the right flanks of the mice and allowed to establish tumors. Tumor volumes (mm^3^) were calculated from the formula 0.5×L×W^2^ (L=length, W=width). All animal experiments were approved by the Ethics Committee of the Shandong University School of Medicine.

**Subcellular fractionation**

Cells were lysed in NP-40 lysis buffer containing 20 mM Tris-HCL (pH 7.9), 150 mM NaCl, 0.5 mM EDTA and 0.5% NP-40 supplemented with protease and phosphatase inhibitors. Lysed cells were kept on ice for 15 min. The lysates were then centrifuged at 2,000 ×g for 5 min. The resulting supernatants represented the cytosolic and membrane fractions. The corresponding pellets representing the nuclear fractions were washed one time in NP-40-containing lysis buffer and sonicated in nuclear lysis buffer (20 mM Tris-HCl (pH 7.4), 450 mM NaCl, 0.5 mM EDTA, 0.5% Triton X-100, 0.1% SDS). The lysates were then centrifuged at 12,000 ×g for 15 min to obtain the cytosolic and nuclear fractions.

**Quantitation of Doc in Subcellular fractions by high-performance liquid chromatography (HPLC)**

Subcellular fractions (cytosolic, nuclear and lysosomal fractions) were isolated from a discontinuous metrizamide density gradient as described with modifications.^6^ Briefly, cells in each sample were treated with Doc or Dox harvested by trypsinization. The cells were centrifuged at 300 ×g for 5 min, resuspended in 0.25 M sucrose and homogenized in a glass Dounce homogenizer. The nuclei were collected by centrifugation at 1000 ×g for 5 min. The supernatant was centrifuged at 12000 ×g for 30 min, and the supernatant was collected as the cytosolic fraction. After resuspension and one wash, the sediment was loaded on top of a discontinuous metrizamide gradient and centrifuged at 65,000 ×g for 2 h. Lysosomes were isolated from a light mitochondrial–lysosomal fraction. Fraction purity was assessed by Western blotting using primary antibodies specific to the following organelle-associated proteins: LAMP2 (lysosomes), GAPDH (cytoplasm), and H3 (nuclear). The cytoplasmic, nuclear and lysosomal supernatants were evaporated to dryness. The residue was analyzed by high-performance liquid chromatography (HPLC) following dissolution in 200 μl MeOH. All HPLC experiments were carried out on an Agilent HPLC system (1260 series, USA). The Doc concentration was positively correlated with the peak area under the standard curve. The fluorescence of Dox in the subcellular fractions was detected at 488/570 nm (excitation/ emission) using multiscan spectrum (Biotek, Cytation5). The concentrations of Doc or Dox in the subcellular fractions were normalized to the protein concentrations.

**Reactive Oxygen Species Assay**

The levels of reactive oxygen species (ROS) in the PC3 and PC3/Doc cells were measured using a 2′7′-dichlorofluorescin diacetate (DCFH-DA) assay kit. Approximately 1×10^6^ cells/well were seeded in 6-well plates. The cells were then suspended in 200 μl of DCFH-DA for 20 minutes at 37°C in the dark. The cells were washed twice with PBS, and the fluorescence intensity was detected by flow cytometry.

**Calcineurin (CaN) activity assay**

CaN activity was assessed using a calcineurin activity assay kit following the manufacturer’s instructions. Then, the protein concentration was determined and used to correct the CaN activity. CaN activity is expressed as a percentage compared with CaN activity in the control group.

**Acidic Sphingomyelinase (ASM) activity assay**

Cells were harvested and rinsed one time using PBS. The cells were then lysed in ice-cold lysis buffer (1% Triton X-100), and the lysates were used immediately for an Acidic Sphingomyelinase (ASM) activity assay. ASM activity was measured with an Amplite™ Fluorimetric Acidic Sphingomyelinase Assay Kit according to the manufacturer's instructions. Specific ASM activity was calculated by normalization to the total protein content.

**Microscopy**
To measure lysosome volumes, cells following various treatments were incubated at 37 °C for 15 min with Lyso-Tracker and Hoechst 33342. The cells were then washed with PBS, and fluorescence derived from aggregated Lyso-Tracker in acidic compartments was measured using confocal microscopy (Carl Zeiss).

**Patient samples**

Prostate cancer, bladder cancer, lung cancer, colon cancer and benign samples were obtained from surgical excision specimens at the Second Hospital of Shandong University, Qilu Hospital Shandong University, and the Shandong Provincial Hospital. Utilization of the clinical samples was approved by the Ethical Committee of the School of Medicine and the Second Hospital of Shandong University.

**Immunohistochemistry (IHC)**

Heat-induced epitope retrieval was performed in 10 mM citric acid buffer (pH 7.2) using a microwave. The slides were incubated at 4 °C overnight with primary antibodies (anti-CTSL, 1:200 dilution; anti-VPS18, 1:500 dilution; anti-LAMP2, 1:200 dilution). An HRP-conjugated antibody and 3,30-diaminobenzidine (DAB) staining were used to visualize primary antibody binding. High-resolution pictures were obtained on a digital electron microscope (NanoZoomer S60), and images were recorded using Case Viewer software. Immunohistochemical results are expressed as a mean score that considers both the intensity of the staining and a positive reaction.

**Transfection**

Cells were transfected with specifically targeted TFEB, TFE3, VPS18, MRP2 (The target sequences are listed in Table S2), and the plasmid of pcDNA3.1, TFEB-Flag, TFE3-Flag, TFE3-GFP, VPS18-Flag, VPS18Δ1-Flag, VPS18Δ2-Flag, VPS18Δ3-Flag, VPS18Δ4-Flag, or VPS18Δ5-Flag using Lipofectamine 2000 Transfection Reagent.

**Drug accumulation and influx**

The efflux and accumulation of the fluorescent probe Rhod-123 and Dox in the cells were determined using flow cytometry. Cells were treated with Rhod-123 or Dox at 37 °C. The cells were collected by centrifugation. After two washes with ice-cold PBS, the cells were resuspended in 0.5 ml of ice-cold PBS and kept on ice for immediate flow cytometry to determine the relative levels of intracellular Rhod-123 or doxorubicin based on fluorescent intensity.

**Statistical analysis**

Western blotting and fluorescent images were analyzed with Image Pro Plus 6.0. The data are presented as the mean ± SD and were analyzed with GraphPad Prism software (GraphPad). Student’s t-test or one-way ANOVA was used for comparisons among different groups. Kaplan-Meier and Cox proportional hazards analyses were used for survival analysis. All the experiments were repeated at least three times. Values of p<0.05 denoted statistical significance and are indicated as *p≤0.05, **p≤0.01, and ***p≤0.001 in the figures.

**Reference**

1. Zhang D*, et al.* Regulation of SOD2 and beta-arrestin1 by interleukin-6 contributes to the increase of IGF-1R expression in docetaxel resistant prostate cancer cells. *European journal of cell biology*. **93**, 289-298 (2014).

2. Wang Y*, et al.* A novel derivative of riccardin D induces cell death through lysosomal rupture in vitro and inhibits tumor growth in vivo. *Cancer letters*. **329**, 207-216 (2013).

3. Hsu JL*, et al.* Phosphodiesterase Type 5 Inhibitors Synergize Vincristine in Killing Castration-Resistant Prostate Cancer Through Amplifying Mitotic Arrest Signaling. *Frontiers in oncology*. **10**, 1274 (2020).

4. Li K, Zhan W, Chen Y, Jha RK, Chen X. Docetaxel and Doxorubicin Codelivery by Nanocarriers for Synergistic Treatment of Prostate Cancer. *Frontiers in pharmacology*. **10**, 1436 (2019).

5. Xu Q*, et al.* Hyper-acetylation contributes to the sensitivity of chemo-resistant prostate cancer cells to histone deacetylase inhibitor Trichostatin A. *Journal of cellular and molecular medicine*. **22**, 1909-1922 (2018).

6. Yu CC*, et al.* A novel small molecule hybrid of vorinostat and DACA displays anticancer activity against human hormone-refractory metastatic prostate cancer through dual inhibition of histone deacetylase and topoisomerase I. *Biochemical pharmacology*. **90**, 320-330 (2014).

**Supplementary Figure Legends and Supplementary Figure**

**Supplementary Figure Legend 1**

(a) GO analysis of organelle-related genes according to cellular component. (b) High-magnification views of organelles in PC3 and PC3/Doc cells by transmission electron microscopy. (c) The mRNA levels of lysosome-related genes were detected with qPCR. (d) Western blotting to detect the levels of lysosome-related proteins in PC3 and PC3/Doc cells. (e) Visualization of intracellular fluorescence for endosome (RAB5), lysosome (LAMP2，Lyso-Tracker) markers in PC3 and PC3/Doc cells. (f) Visualization of the intracellular fluorescence of Lyso-Tracker (in red) and DAPI (in blue) in PC3 cells treated with Doc (12 nM). (g) Western blotting analysis of metabolism-related proteins in several parental cells and their drug resistant counterparts. (h) Visualization of the intracellular fluorescence of Lyso-Tracker (in red) and DAPI (in blue) in several parental cells and their drug resistant counterparts. (i) Model of the animal experiment. (j) Photographs of excised tumors from five groups are shown. Doc (5 mg/kg), Dox (2 mg/kg), VCR (0.5 mg/kg), VP16 (10 mg/kg). (k) Heat maps of lysosome-related gene expression in drug resistant animal models were determined with qPCR. (l) Immunohistochemistry showed the high-level expression of lysosome-related proteins in drug resistant animal models. Data are the mean ± SD; *p < 0.05, **p < 0.01 and ***p < 0.001.

**Supplementary Figure Legend 2**

(a) TFEB or TFE3 were knocked down with siRNA, and the expression of lysosome-related genes was analyzed. (b) TFEB or TFE3 were knocked down with siRNA, and cells were stained with Lyso-Tracker (in red) and DAPI (in blue). (c) The expression of lysosome-related genes was analyzed following the overexpression of TFE3 or TFEB. (d) Western blotting analysis of the nuclear translocation of TFEB and TFE3 in 293T cells incubated with Doc (12 nM) for different lengths of time. (e) The nuclear translocation of TFE3 in drug resistant animal models was detected by immunohistochemistry. (f) Flow cytometry analysis of ROS in PC3 and PC3/Doc cells. (g) Differences in the expression of several kinases between PC3 and PC3/Doc cells were monitored by western blotting. (h) The expression of calcineurin subunit A (CaN A) was detected by western blotting. (i) The activity of CaN was detected in PC3 and PC3/Doc cells. (j) Immunoblots showing the TFE3 state in nuclear and cytosolic fractions of 293T cells incubated with Doc (12 nM) and FK520 (0.5 μM), a calcineurin inhibitor. Data are the mean ± SD; *p < 0.05, **p < 0.01 and ***p < 0.001.

**Supplementary Figure Legend 3**

(a) siRNA knockdown TFE3 was performed in combination with Doc and Dox treatment, and the survival rate was analyzed. (b) Cells overexpressing TFE3 underwent combination treatment with Doc and Dox. (c) Flow cytometry analysis of the accumulation of Rhodamine (Rho, 1 μM) in PC3 and PC3/Doc cells. (d) Flow cytometry analysis of the accumulation of Dox (0.8 μM) in PC3 and PC3/Doc cells. (e) Flow cytometry analysis of the influx of Rho (1 μM) following the knockdown of TFE3. (f) Flow cytometry analysis of the influx of Dox (0.8 μM) following the knockdown of TFE3. (g) Flow cytometry analysis of the influx of Rho (1 μM) following the overexpression of TFE3. (h) Flow cytometry analysis of the influx of Dox (0.8 μM) following the overexpression of TFE3. (i) Typical markers served as indicators of every organelle fraction (cytoplasm (Cyto), nuclear (Nuc) and lysosome (Lyso)) and were detected with western blotting. (j) Distribution of Dox in different organelles between PC3 and PC3/Doc cells. (k) The Doc concentration was measured by high-performance liquid chromatography (HPLC). Data are the mean ± SD; *p < 0.05, **p < 0.01 and ***p < 0.001.

**Supplementary Figure Legend 4**

(a) Gene expression profiling revealed that most ABC superfamily efflux transporters were increased in drug-resistant cells. (b) Some ABC superfamily efflux transporters were detected by qPCR. (c) The expression of MRP2 was detected by western blotting. (d) siRNA knockdown of MRP2 was performed in combination with Doc and Dox treatment, and the survival rate was analyzed. (e) Changes in the distribution of Dox in different organelles (cytoplasm (Cyto), nuclear (Nuc) and lysosome (Lyso)) of TFE3-overexpressing cells. (f) Knockdown of MRP2 and/or TFE3-overexpressing were performed in combination with Doc and Dox treatment, and the survival rate was analyzed. (g) The E-box motif of MRP2. (h) siRNA knockdown of TFE3 was performed in combination with Doc (12 nM) and Dox (0.8 μM) treatment, and the expression of *ABCC2* was analyzed by qPCR. (i) Cells in which TFE3 was knocked down with siRNA or TFE3-overexpressing were treated with a combination of Doc (12 nM) and Dox (0.8 μM), and the expression of *TFE3* was analyzed. Data are the mean ± SD; *p < 0.05, **p < 0.01 and ***p < 0.001.

**Supplementary Figure Legend 5**

(a) Survival rate of PC3 and PC3/Doc cells treated with Doc, RDN, hydroxychloroquine (HCQ) and salinomycin (SAL). (b) Visualization of intracellular fluorescence from Lyso-Tracker (in red) in PC3/Doc cells treated with RDN (2 μM) and hydroxychloroquine (HCQ) (25 μM). (c) Western blotting analysis of changes in autophagy-related proteins following treatment with RDN (2 μM). (d) Change of acid sphingomyelinase (ASM) activity following treatment with RDN (2 μM). HCQ (25 μM) was used as a positive control. (e) Lysosome maturation was analyzed by a pH-sensitive RFP-GFP-LC3 reporter plasmid following treatment with RDN (2 μM). Rapa (2 μM) was used as a positive control. (f) The expression of MRP2 was detected by western blotting following treatment with RDN (2 μM). (g) Distribution of MRP2 and its colocalization with LAMP2 in PC3/Doc cells in combination with RDN (2 μM) treatment. (h) Tumors treated with placebo (Ctrl), RDN (20 mg/kg), and Doc (5 mg/kg) were quantified using bioluminescence imagining. Significant changes in bioluminescence intensity (photon flux: photon/s/cm^2^/square root) between control and experimental mice. (i) Biochemical analysis of liver and renal function, aspartate transaminase (AST), glutamic-pyruvic transaminase (ALT), blood urea nitrogen (BUN), and creatinine (CREA). (j) Tumor weights from three groups are shown. (k) The body weights of mice in different groups were recorded every 3 days. (l) Survival rate of PC3/Doc cells treated with RDN and RDN-Bio. (m) Visualization of the intracellular colocalization of RDN-biotin (1 μM) and LAMP2 by confocal microscopy. Data are the mean ± SD; *p < 0.05, **p < 0.01 and ***p < 0.001.

**Supplementary Figure Legend 6**

(a) The expression of VPS18 in prostate cancer (PRAD), bladder cancer (BLCA) and liver cancer (LIHC) and adjacent noncancerous tissues was detected by immunohistochemistry. (b) VPS18 is correlated with poor prognosis in several human tumors. (c) shRNA knockdown of VPS18. (d) Cell growth were analyzed using a xCELLigence RTCADP instrument following shRNA knockdown of VPS18. (e) Cell growth were analyzed using [clone](javascript:;) [formation](javascript:;) [assay](javascript:;) following shRNA knockdown of VPS18. (f) Tumor weights from two groups are shown. (g) The expression of VPS18 and Ki67 staining in animal models was detected by immunohistochemistry. (h) TFE3-overexpressing cells were treated with Doc (12 nM), and the expression of *VPS18* was analyzed by qPCR. (i) The expression of VPS18 in drug resistant animal models was detected by immunohistochemistry. (j) The expression of VPS18 in several parental cells and drug-resistant cells was detected by western blotting. (k) shRNA knockdown of VPS18 was performed in combination with Doc and Dox treatment, and the survival rate was analyzed. (l) VPS18-overexpressing cells were treated with Doc and Dox, and the survival rate was analyzed.

**Supplementary Figure 1**


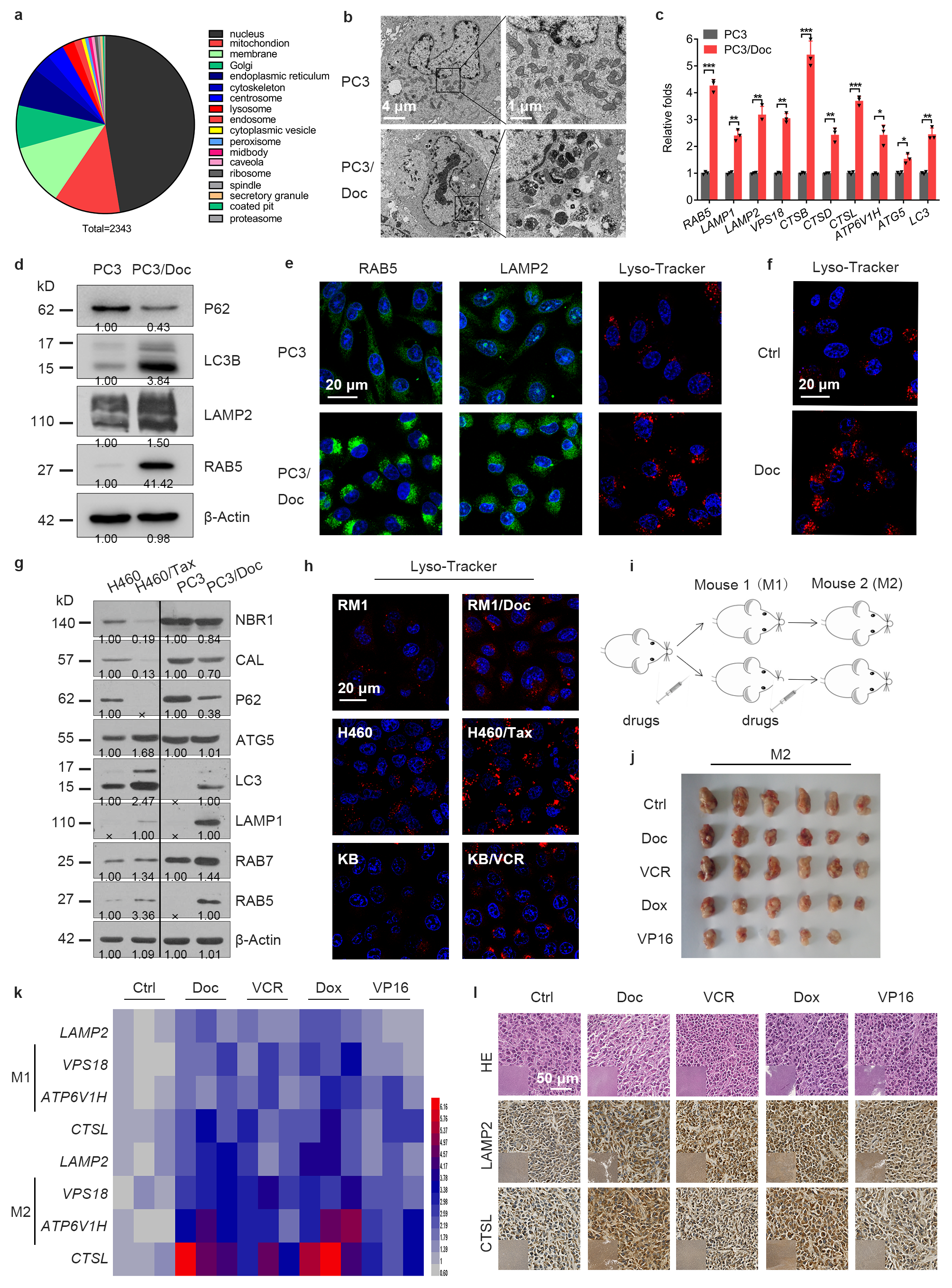


**Supplementary Figure 2**


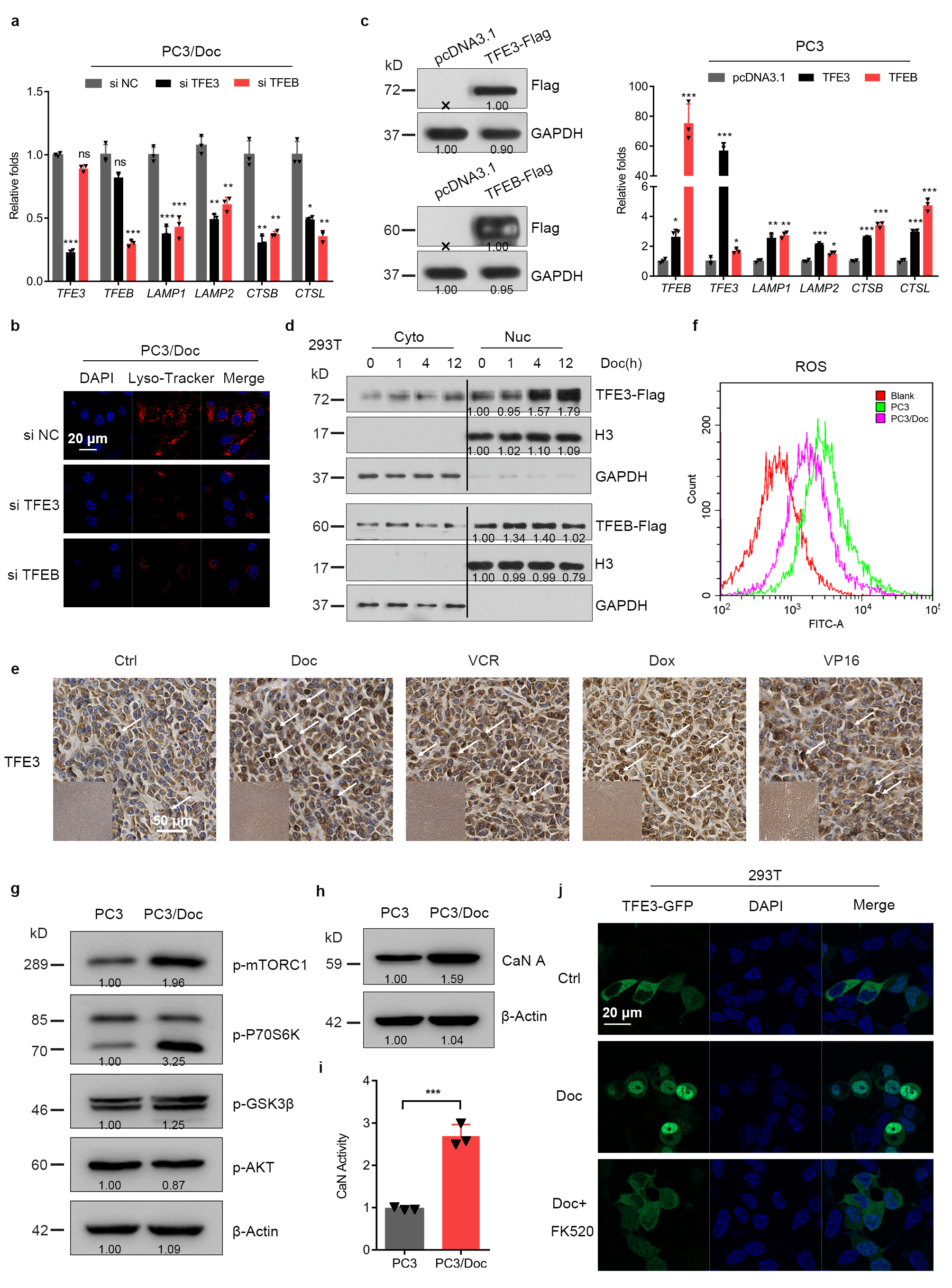


**Supplementary Figure 3**


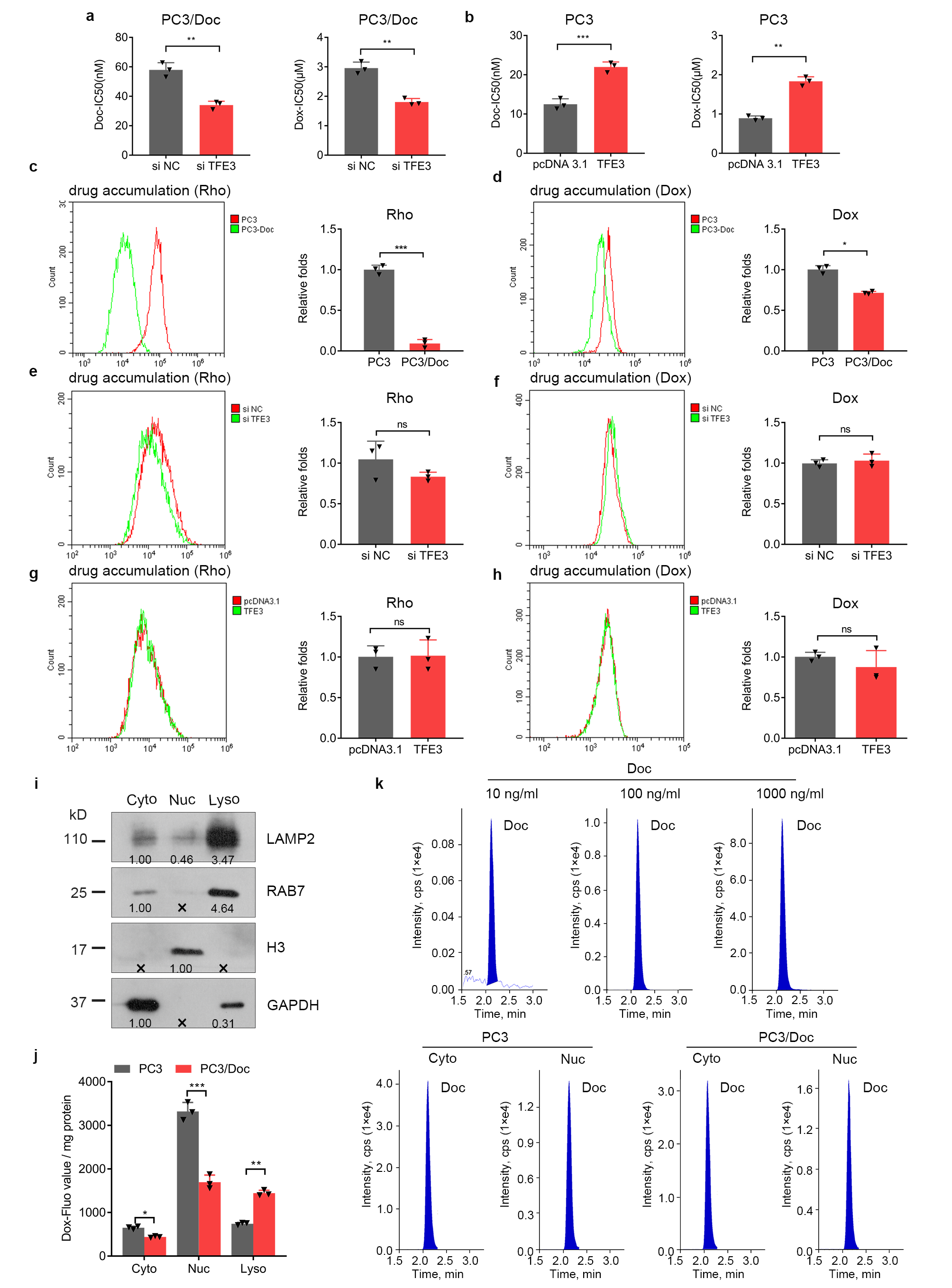


**Supplementary Figure 4**


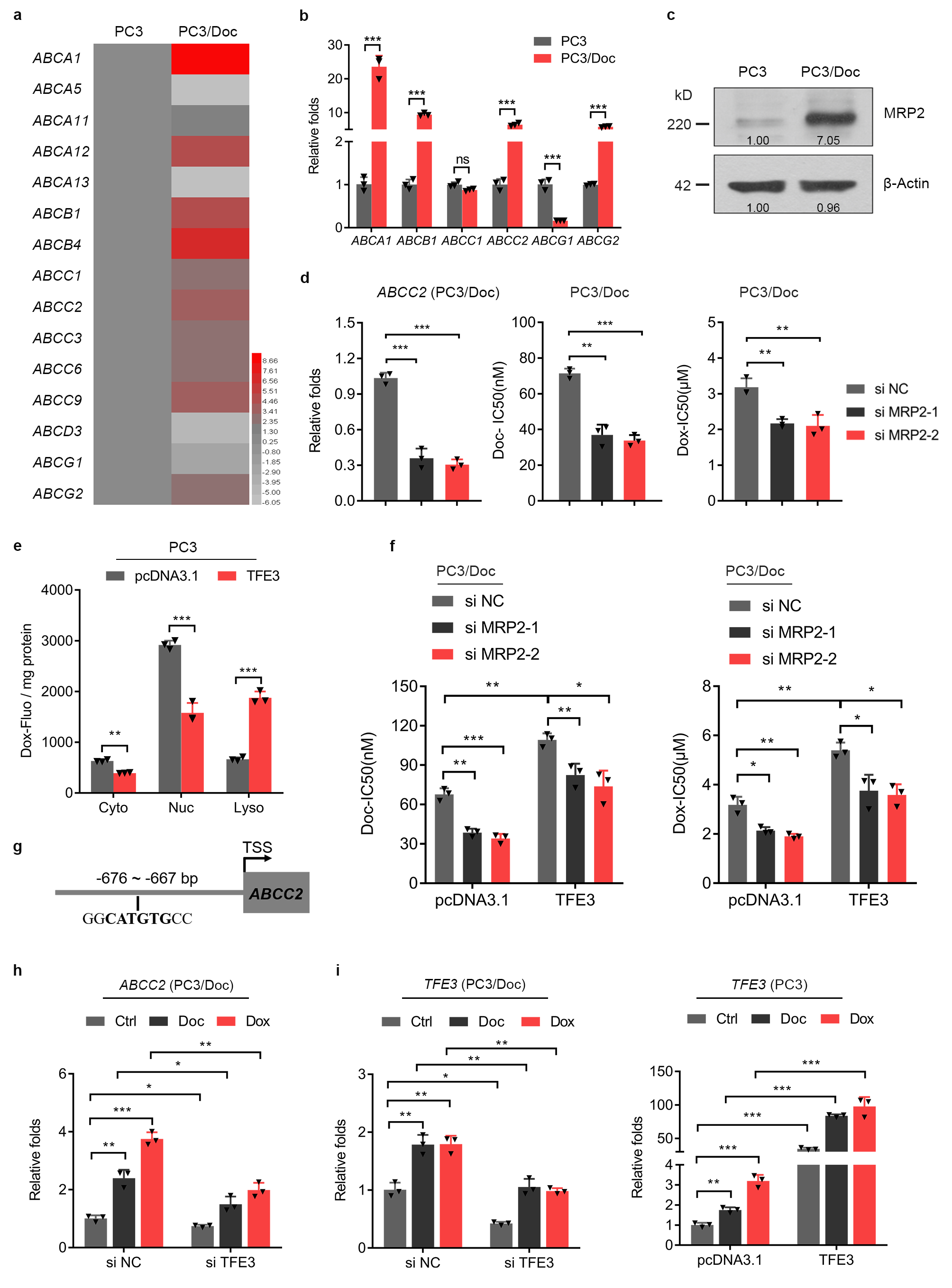


**Supplementary Figure 5**


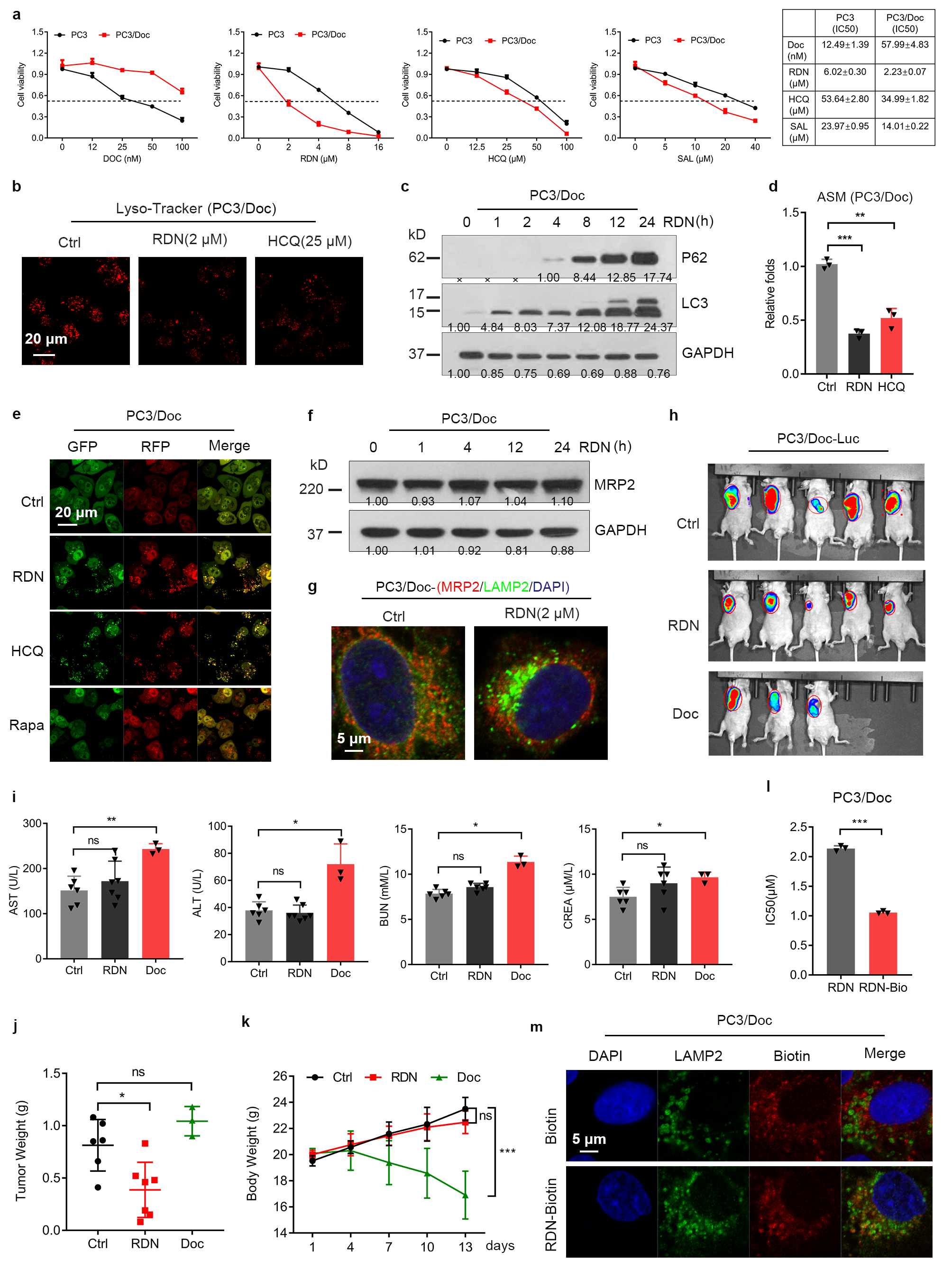


**Supplementary Figure 6**


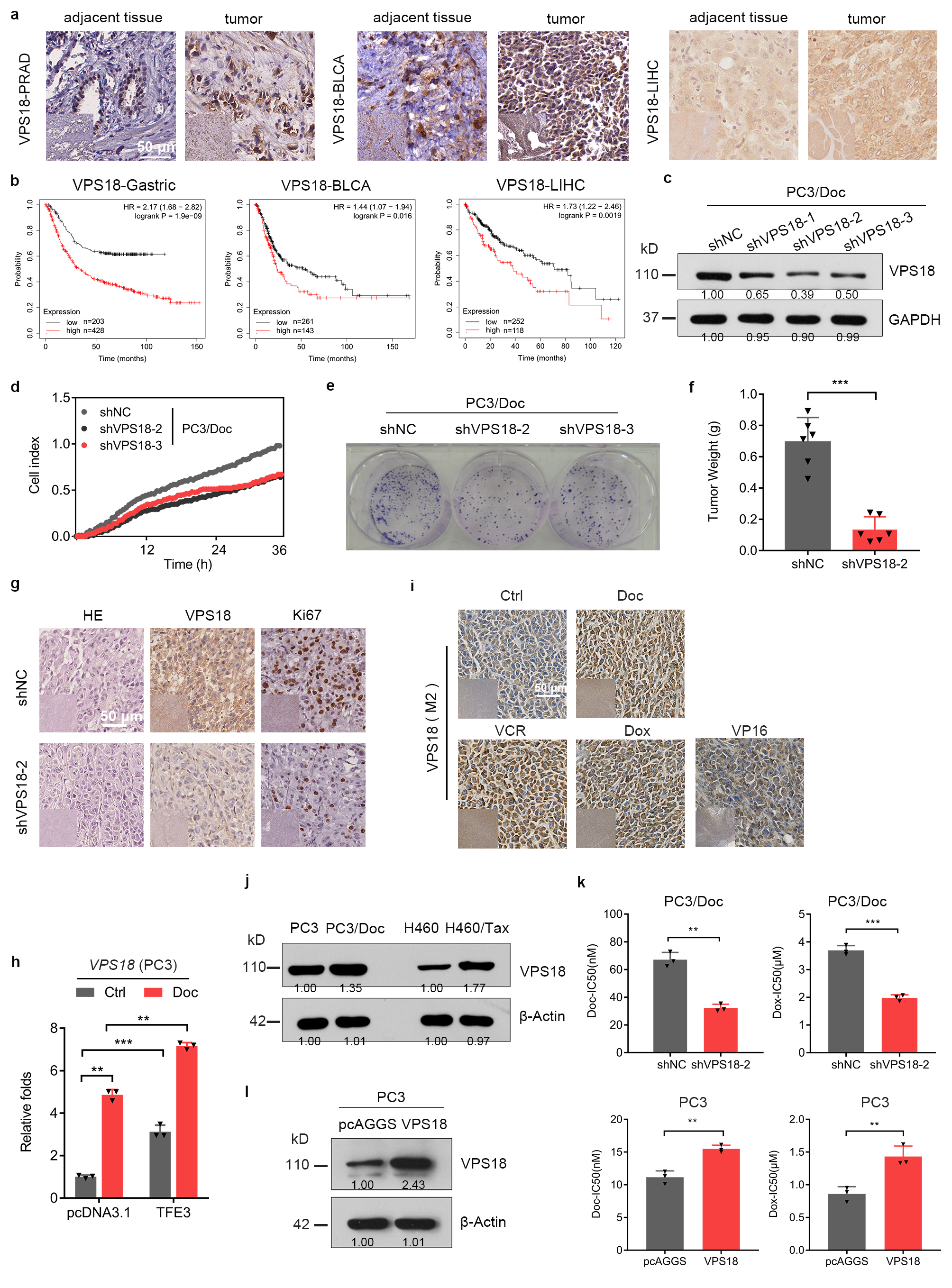


**Supplementary Table 1:**

| Name (Human) | [Forward](javascript:;) [primer](javascript:;) | Reverse primer |
| --- | --- | --- |
| *RAB5* | AGACCCAACGGGCCAAATAC | GCCCCAATGGTACTCTCTTGAA |
| *LAMP1* | ACGTTACAGCGTCCAGCTCAT | TCTTTGGAGCTCGCATTGG |
| *LAMP2* | TGGCAATGATACTTGTCTGCTG | ACGGAGCCATTAACCAAATACAT |
| *VPS18* | CACTCGGGGTATGTGAATGCC | TCGGAAGGGGTGAAGTCAATG |
| *CTSB* | AGTGGAGAATGGCACACCCTA | AAGAAGCCATTGTCACCCCA |
| *CTSD* | TGCTCAAGAACTACATGGACGC | CGAAGACGACTGTGAAGCACT |
| *CTSL* | CTTTTGCCTGGGAATTGCCTC | CATCGCCTTCCACTTGGTC |
| *ATP6V1H* | CAGAAGTTCGTGCAAACAAAGTC | TCAGGGCTTCGTTTCATTTCAA |
| *ATG5* | AGAAGCTGTTTCGTCCTGTGG | AGGTGTTTCCAACATTGGCTC |
| *LC3B* | AAGGCGCTTACAGCTCAATG | CTGGGAGGCATAGACCATGT |
| *TFE3* | CCGTGTTCGTGCTGTTGGA | GCTCGTAGAAGCTGTCAGGAT |
| *ABCA1* | ACATCCTGAAGCCAATCCTGA | CTCCTGTCGCATGTCACTCC |
| *ABCB1* | GGGAGCTTAACACCCGACTTA | GCCAAAATCACAAGGGTTAGCT |
| *ABCC1* | CTCTATCTCTCCCGACATGACC | AGCAGACGATCCACAGCAAAA |
| *ABCC2* | CCCTGCTGTTCGATATACCAATC | TCGAGAGAATCCAGAATAGGGAC |
| *ABCG1* | ATTCAGGGACCTTTCCTATTCGG | CTCACCACTATTGAACTTCCCG |
| *ACCG2* | ACGAACGGATTAACAGGGTCA | CTCCAGACACACCACGGAT |
| *VPS11* | CAATCCACTCTGCACTCGAAT | CGGGTGATGTCTCCTTTGTTCA |
| *VPS16* | TACACGGCGAACTGGAACC | GCCTCACACTAGCAGCTTTCT |
| *VPS33A* | ATGGCGGCTCATCTGTCCTA | CATCCCAAACTATTGCCTTGCT |
| *β-Actin* | CATGTACGTTGCTATCCAGGC | CTCCTTAATGTCACGCACGAT |
| *GAPDH* | GGAGCGAGATCCCTCCAAAAT | GGCTGTTGTCATACTTCTCATGG |
| *Name (Mouse)* | [Forward](javascript:;) [primer](javascript:;) | Reverse primer |
| *LAMP2* | TGTATTTGGCTAATGGCTCAGC | TATGGGCACAAGGAAGTTGTC |
| *VPS18* | AGTACGAGGACTCATTGTCCC | TGGGCACTTACATACCCAGAAT |
| *CTSL* | ATCAAACCTTTAGTGCAGAGTGG | CTGTATTCCCCGTTGTGTAGC |
| *ATP6V1H* | GGATGCTGCTGTCCCAACTAA | TCTCTTGCTTGTCCTCGGAAC |
| *β-Actin* | GGCTGTATTCCCCTCCATCG | CCAGTTGGTAACAATGCCATGT |

**Supplementary Table 2**

|  | The specifically targeted sequence |
| --- | --- |
| TFEB | AGACGAAGGUUCAACAUCA |
| TFE3 | CGCAGGCGATTCAACATTAAC |
| VPS18-1 | TCTGGCGCACCTATCTGGACATGAA |
| VPS18-2 | GAATGCTTTCGAACCTTCCTCAGCA |
| VPS18-3 | TATCCATGAGCTGCTCGCCAGTCAT |
| MRP2-1 | CCAGCAAAGGCAAGAUCCAGUUUAA |
| MRP2-2 | GAUCAUGAAUGAGAUUCUUAGUGG |
| MRP2-3 | ACCAAGACAUUAGUGAGCAAGUUUG |
